# Neural correlates of error prediction in a complex motor task

**DOI:** 10.3389/fnbeh.2015.00209

**Published:** 2015-08-05

**Authors:** Lisa Katharina Maurer, Heiko Maurer, Hermann Müller

**Affiliations:** Neuromotor Behavior Lab, Department of Psychology and Sport Science, Justus-Liebig-University GiessenGiessen, Germany

**Keywords:** error prediction, error-related negativity, motor task, ballistic throwing task, forward modelling, electroencephalography

## Abstract

The goal of the study was to quantify error prediction processes via neural correlates in the Electroencephalogram (EEG). Access to such a neural signal will allow to gain insights into functional and temporal aspects of error perception in the course of learning. We focused on the error negativity (Ne) or error-related negativity (ERN) as a candidate index for the prediction processes. We have used a virtual goal-oriented throwing task where participants used a lever to throw a virtual ball displayed on a computer monitor with the goal of hitting a virtual target as often as possible. After one day of practice with 400 trials, participants performed another 400 trials on a second day with EEG measurement. After error trials (i.e., when the ball missed the target), we found a sharp negative deflection in the EEG peaking 250 ms after ball release (mean amplitude: *t* = −2.5, *df* = 20, *p* = 0.02) and another broader negative deflection following the first, reaching from about 300 ms after release until unambiguous visual knowledge of results (KR; hitting or passing by the target; mean amplitude: *t* = −7.5, *df* = 20, *p* < 0.001). According to shape and timing of the two deflections, we assume that the first deflection represents a predictive Ne/ERN (prediction based on efferent commands and proprioceptive feedback) while the second deflection might have arisen from action monitoring.

## Introduction

Prediction plays a role at many different levels in motor control. Athletes, for instance, are experts in predicting movement outcomes in their sport. Basketball players can rate whether a free throw will be successful or not even shortly after the ball leaves their hand. But also in everyday live, we constantly predict the outcome or the process of our movements. Prediction is the reason why we cannot tickle ourselves because we know the sensory effect in advance and are therefore not overtaken by the action (Weiskrantz et al., 1971). In addition, predictions can also alert us to erroneous performance and the need for adaptations to changing task demands and environmental circumstances. By means of electroencephalography (EEG), more concretely with event-related potentials, it is possible to observe error processing in the brain. Among these potentials, the Ne/ERN is a negative deflection in fronto-central regions that emerges with or shortly after an erroneous motor response, typically in speeded choice reaction time tasks (e.g., flanker task), prior to sensory feedback about the movement outcome such as knowledge of results (KR; Falkenstein et al., 1991; Gehring et al., 1993). Different theoretical accounts of the Ne/ERN have explained its functional significance with reference to error detection (i.e., the comparison of expected/planned and actual outcomes; Falkenstein et al., 1991), response conflict (Botvinick et al., 2001), and reinforcement learning (Holroyd and Coles, 2002), respectively. In the absence of KR however, all of these possible functions of the processes shining up in the Ne/ERN necessarily require a prediction about the course and/or outcome of an action in order to detect deviations from an intended course of action, or to evaluate a conflict between concurrently active responses. Based on the emergence of the Ne/ERN prior to the availability of KR, it is reasonable to assume that the Ne/ERN makes use of the predictive output of the central nervous system.

There are studies supporting these assumptions. In an auditory-motor task (re-producing piano tones) Lutz et al. (2013) found that the Ne/ERN amplitude of participants increased during learning, whereas another EEG error component occurring after provision of feedback did not exhibit any practice-related changes. The authors argued that while participants learned the auditory-motor mapping of the task, prediction of their movement effects became increasingly reliable and that these predictions finally manifested in the Ne/ERN signal when they conducted an error. At the end of the experiment, the Ne/ERN was larger in self-inflicted errors, which could be predicted, relative to externally evoked errors, which could not be predicted in this experiment. Moreover, Peterburs et al. (2012) could show that in an antisaccade task the Ne/ERN of cerebellar patients was significantly reduced compared to healthy persons. They conclude that the cerebellum, which is conceived to house predictive forward models in its microstructures (Miall and Wolpert, 1996; Miall, 1998; Wolpert et al., 1998), contributes to the generation of the Ne/ERN. Studies from the group of Holroyd and colleagues add to these findings. In a series of similar experiments where a specific stimulus-response mapping had to be learned, the authors could show that the Ne/ERN emerges after an erroneous response, but prior to external feedback (Holroyd and Coles, 2002; Nieuwenhuis et al., 2002), and that the anterior cingulate cortex (source of Ne/ERN) is active when only internal information about a conducted error is available (Holroyd et al., 2004).

However, the predictions the system has to make in the described studies are more of a cognitive nature as they primarily refer to the outcome of an action (thus evaluating whether the decision on WHAT action should be selected was correct) as opposed to predictions about kinematics and dynamics of movements (thus evaluating HOW a particular action is executed). In the literature we can also find ERP studies using explicit motor tasks that, nonetheless, are differently located on that WHAT-HOW-scale. One can distinguish between goal attainment errors (“high-level” errors; Krigolson and Holroyd, 2006) such as one’s failure to achieve an intended action goal and execution errors ("low-level” errors; Krigolson and Holroyd, 2006) i.e., deviations between actual and desired movement kinematics. Especially Krigolson and Holroyd have suggested that the Ne/ERN is elicited when “high-level” errors are made, for instance, when participants fail to reach a target (Krigolson and Holroyd, 2007a; Krigolson et al., 2008), respond incorrectly with the wrong hand or force level (de Bruijn et al., 2003), or avoid a collision with a non-target (Krigolson and Holroyd, 2006, 2007b). Whereas “low-level” errors should be rather associated with activity in posterior parts of the brain. Studies addressing “low-level” errors are, however, not that clear in their results. When participants deviated from a designated movement trajectory, Ne/ERN like fronto-central activity was found by Krigolson and Holroyd (2006, 2007a,b). When, however, the trajectory deviation was correctable, negative activation in posterior parietal cortex (Krigolson and Holroyd, 2007a; Krigolson et al., 2008) as well as medial-frontal activity (Anguera et al., 2009) was found. These rather contradictory results might be explained by methodological differences between the studies. Beyond that, one has to note that all tasks had an overall action goal (reaching for, or staying on a target). Hence, an error in movement execution (“low-level”) can lead to a “high-level” error. This “high-level” error can be predicted in case corrections are expected to fail. We, therefore, propose that it is not only the actual failure to achieve a particular action goal, but also the anticipated failure that can give rise to a fronto-central error negativity (Ne) in the frontal “high-level” error system. The anticipation, in that case, is based on predictive systems using internal and external information about the movement.

Since in all tasks of the presented studies the prediction about the action goal takes place while the movement is still executed and therewith coincides with potential corrective movements [whether they were possible (Anguera et al., 2009) or not (Krigolson and Holroyd, 2007b)]. Thus, execution and the correction process, respectively, might also contribute to the Ne and the Ne/ERN-signal could not solely be attributed to prediction processes. Hence, for any generalizable conclusions with respect to sensorimotor prediction it is necessary to transfer the current findings to a task that allows separating these processes. Ballistic goal-oriented throwing tasks are suitable for this purpose because, here, predictions about task success or failure (hit vs. miss, “high-level”) are based on relatively complex information processing from different sources (i.e., motor commands, proprioceptive feedback) about movement execution (“low-level”). In addition, ballistic tasks are too fast for online corrections and throwing tasks, in particular, allow no corrections after release. Moreover, the KR in such tasks is delayed with respect to movement termination due to the flight time of the object to be thrown.

Here, we used a virtual goal-oriented throwing task where participants had to throw a virtual ball displayed on a computer monitor with the aim to hit a virtual target as often as possible by manipulating a real lever. After error trials (i.e., when the ball missed the target), we found two Ne/ERN like negative deflections in the EEG signal that are discussed in light of error prediction as well as feedback processing.

## Materials and Methods

### Participants

Twenty-nine participants (six males) with a mean age of 23.2 (*SD* = 6.3) were tested. They had normal or corrected-to-normal vision and performed all trials with the right hand, irrespective of whether this was their dominant or non-dominant hand (for explanation see below). They gave written informed consent in accordance with the Declaration of Helsinki and the protocol was approved by the Ethical Review Board of the Justus-Liebig-University, Giessen.

### Task and Apparatus

**Figure 1:**
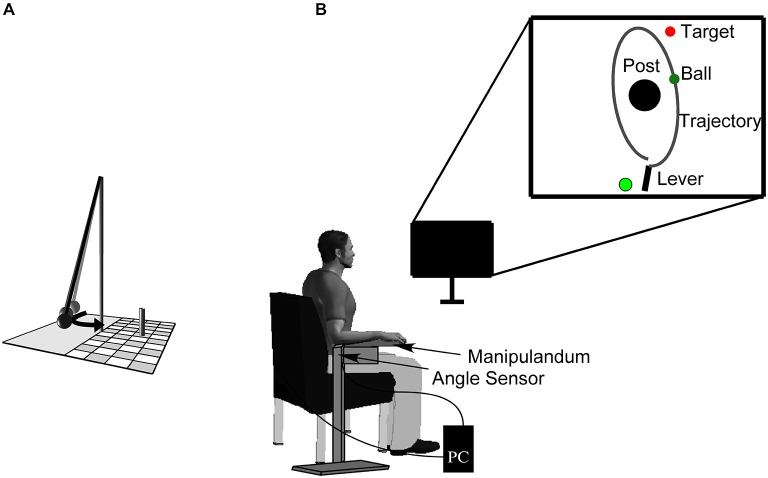
**Sketch of the real throwing task (A)**. A ball is suspended on a string and swings around the center post, with the objective of knocking down the skittle at the opposite side. Experimental set-up **(B)**. Participants operate a manipulandum to throw the virtual ball on the monitor in front of them with the goal to hit the target located behind the center post. The circle to the left of the lever represents the position from where participants have to start the movement (it starts out to be red and then turns to green signaling participants that they are free to move). The angular displacement of the participant’s forearm is measured by a magnetic angle sensor and recorded by the computer.

The experimental task was a semi-virtual, goal-oriented throwing task where participants had to hit the goal in series as often as they could. Monetary rewards of 30, 20, and 10 € were given to the subjects achieving the three longest hit series. The throwing task, that has been used in other studies (e.g., Müller and Sternad, 2004; Maurer et al., 2011, 2014; Pendt et al., 2011), is based on a British pub game in which a ball is suspended from a string attached to the tip of a vertical post. The player has to throw the ball around the post in order to knock down a target skittle on the other side (Figure 1). The movements of the participants in the experimental task were real, whereas the ball flight was virtual. Sitting frontal to the computer screen, participants rested their forearm on a metal lever (the manipulandum) with a metal support padded with foam rubber. They saw the work space of the task, including a virtual version of the manipulandum, in two dimensions from a bird’s eye view on a computer screen from which they sat approximately 1 m away. The post in the center of the work space was represented by a cone of 25 cm radius at position *x* = 0.0 m, *y* = 0.0 m. A circular target of 5 cm radius was presented with its center 35 cm to the right and 100 cm above the center of the post. The virtual lever was represented as a solid bar of 40 cm length, fixed at one end (Figure 1), corresponding to the real horizontal manipulandum that was fixed to a vertical support and pivoted around an axle centered directly underneath the elbow joint. The manipulandum could be adjusted to a comfortable height for each participant. Rotations of the manipulandum were measured by a magnetic angle sensor (resolution 12 bit, 0.09 deg) with a sampling rate of 1000 Hz.

To hold the virtual ball with the virtual lever on the display, a switch at the free end of the metal lever had to be touched by the participant. Upon releasing the contact, an electrical current was disrupted and this accounted as trigger for releasing the virtual ball. To throw, participants first closed the switch with their index finger, then rotated the forearm in an outward horizontal motion and simultaneously released the switch. The ball traversed on a trajectory initialized by the angle and velocity of the participant’s arm at the moment of release. Both the movements of the virtual lever and the simulated trajectory of the ball were displayed on the screen in real time. The ball’s trajectory was determined by the simulated physics of the task and described an elliptic path around the pole (Müller and Sternad, 2004). The task itself has no unique solution to achieve the goal providing an additional challenge to select a solution (Müller and Sternad, 2009). Additionally, the center post between lever and target impeded trivial solutions, i.e., releasing with zero velocity. The target was hit by the ball when the minimal distance between ball trajectory and center of the target was less than 10 cm. The result measure was defined as the number of subsequent target hits (hit series).

### Experimental Design and Procedure

Participants performed the task on two days. The first day served as practice. On the second day, EEG-recording was conducted. On both days, the session was terminated with the first error after the 400th trial. Therefore, total number of trials could vary between participants. Participants were instructed to throw the ball with their right hand in a counter-clockwise trajectory around the center post in order to hit the target in series as often as possible. Movement direction was clockwise, similar to performing a Frisbee backhand. Due to technical reasons we also instructed left-handed participants to throw with the right hand. We did not expect that this affected our results since the task is easy to perform with the non-dominant hand and if left-handers should indeed, for instance, show a poorer error prediction, we would expect a conservative error and no fundamental distortion of results.

The release signal of the electrical switch at the manipulandum was forwarded to the EEG-amplifier and used as synchronization trigger “release”. An additional trigger “feedback” was exported at the moment of minimal distance between ball trajectory and target. Time between release and this KR feedback varied minimally between trials of individual participants and ranged from 817 to 910 ms over all participants (dependent on throwing strategy).

To prevent a fast rhythmic execution of throws a red-colored starting light emerged at the beginning of each trial (see Figure 1). Participants were instructed to move the virtual lever with the ball at its end into the red circle that thereby changed to yellow. After 1 s of holding the ball steady in the circle, it turned green, after which participants were free to move. Note that they were not instructed to throw in reaction to the signal. By this procedure, time between release of one trial and the beginning of the subsequent one was on average 2.2 s. A trial was considered valid when, after the green signal, the manipulandum exceeded an angular velocity of 50°/s followed by the ball release.

### EEG-Recordings and Data-Preprocessing

For EEG and electrooculogram (EOG) recordings, we used a 16 channel AC/DC amplifier with Ag/AgCl active scalp electrodes (V-Amp, Brain Products). Electrodes were placed with an electrode cap (actiCap, Brain Products) according to the international 10–20 system on F3, Fz, F4, FCz, C3, Cz, C4, P3, Pz, P4. The ground electrode was placed on Fpz, the online reference electrode on the left mastoid (to avoid artifacts from right-hand movements). A second reference electrode was located on the right mastoid which was used for offline re-referencing by means of averaged mastoids. Eye electrodes were placed on the external canthi of both eyes as well as above and below the right eye to measure horizontal and vertical eye movements. Being an active electrode system, electrode impedance was kept below 25 kΩ. The data were digitized at a sampling rate of 500 Hz. EEG and EOG were filtered online with a highpass filter at 0.01 Hz. EEG data was additionally filtered offline with a phase-shift free Butterworth filter (0.2–30 Hz bandpass). Ocular artifacts were removed from continuous EEG signal using Infomax Independent Component Analysis (Makeig et al., 1996, 1997). EEG was segmented into epochs around the release trigger, beginning 600 ms before “release” and ending 1000 ms after “release”. The segments were baseline corrected by subtracting the average amplitude of the complete signal. Single segments were then additionally visually inspected in order to remove epochs that contain artifacts. Since the time of KR (when the ball hits or misses the target) in relation to release differed between participants, we additionally re-referenced the EEG epochs with respect to the trigger “feedback” and created segments starting 1000 ms before and 800 ms after KR feedback.

### Behavioral Data Processing—Classification of Errors and Successful Trials

In the experimental task an error was defined when the ball missed the target since the goal for the participants was to subsequently hit the target as often as possible. The trials in the *error* category were expected to produce an at least larger error signal in the EEG relative to the trials in the *hit* category. The ball missed the target when the minimal distance of its trajectory to the target center was larger than 10 cm (because the ball’s and target’s radius was 5 cm each). The two classes of result (“hit”, “error”) were clearly discriminable for the subject since only in case of a hit, a collision sound was presented and the target ball was knocked out of its position. To allow for a better discrimination between “hits” and “errors” in the analyses we excluded marginal hits and misses close to this 10 cm-distance. The distances, however, varied between participants, i.e., some subjects had many central hits with zero or close to zero distance and marginal misses while others had more marginal hits and misses with large distances instead. Hence, we did not define a fixed transition value from hits to misses but rather created individual categories. Firstly, we sorted the trials with respect to distance within each participant. Secondly, we classified up to 50 trials of the lower end of the distance vector with a distance smaller than 5 cm as hits and up to 50 trials with a distance larger than 12 cm as errors. During artifact rejection, a varying number of segments were removed for individual participants. Five participants, where the procedure did not yield at least 20 trials per category, were excluded from all further analyses.

As an additional behavioral measure, we determined the relative number of total target hits (not necessarily in series) in percent for all participants in order to be able to discriminate between participants with respect to task performance. We used this measure as opposed to the longest hit series because the hit series is very much dependent on other variables like noise or arousal (e.g., due to a possible higher performance pressure with increasing hit series length). We observed that some participants differed strongly from all others with respect to this performance criterion. In addition, we need to point out that the release angle and its dispersion are crucial determinants of performance. Performers who yield high numbers of hits are typically quite constant regarding their release angle (one can say, they know “where” to throw). In contrast, performers with a high dispersion in release angle have not found an adequate throwing strategy, i.e., they still search and try different angles. We can assume that such participants vary their throwing strategy unusually and any correlation between neural activation and error might be grounded on a different basis in these participants relative to all others. Thus, we also checked differences between participants with respect to angle dispersion. Participants who deviated at least ±2 standard deviations in performance (relative number of total target hits) or release angle dispersion were dismissed from further analyses. In consequence, we discarded data sets of three more participants.

### Analysis of Error Related Signals

EEG epochs of *error* trials and *hits* trials were averaged for each participant. Then, a difference wave between the two categories was computed. The Ne/ERN is typically found at the 10–20 location FCz. We, therefore, chose to perform all further analyses on activity recorded at the FCz electrode. Since this is, to our knowledge, the first study to analyze error related signals in a ballistic throwing task, we could not predefine a time window after release where the occurrence of such a signal would be expected. Hence, we used the grand average of the difference wave to define the time window over which we then computed the mean amplitude of the difference waves for each participant. With a one-sample *t*-test we tested whether the mean amplitude deviated negatively from zero. Since task performance differed strongly between participants, we tested a possible influence on error prediction by correlating the percentage of target hits with the error signal amplitude. Results were regarded as significant when *p* < 0.05.

## Results

### Behavioral Results

Over all participants, we yielded on average 39.6 target hits and 33.8 errors for the grand average. With respect to performance, there was a large difference between participants. On average, they hit the target in the second (the experimental) session in 75.1% of trials (*SD* = 13.1%). The range was between 48.1 and 95.7%. Note that the total number of trials also varied between participants since the experiment was only terminated after the last ongoing hit series exceeding 400 trials. Here, the range was between 400 and 437. The total number of trials did, however, not correlate with the number of target hits (*r* = 0.17, *p* = 0.41).

### Electrophysiological Results

**Figure 2:**
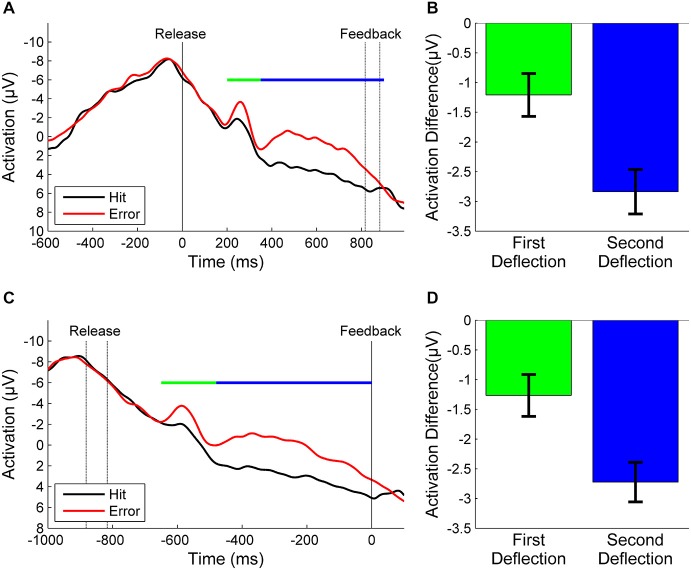
**(A)**: Grand averages of *error* trials (red) and *hit* trials (black) synchronized to the moment of release (motor response) and **(B)**: the corresponding mean amplitudes of the difference waves between *errors* and *hits* for the first and second deflections. **(C)**: Grand averages synchronized to the moment of KR-feedback and **(D)**: corresponding mean amplitudes. The time windows over which the mean amplitudes were calculated are marked by a green line for the first deflection and a blue line for the second deflection, respectively. The time between release and KR-feedback differed between participants due to different throwing strategies and velocities. To avoid false interpretations due to temporal smearing we re-synchronized the EEG epochs with respect to feedback (see text). In consequence, in the grand average the non-synchronized event [feedback in **(A)**, release in **(C)**] varies in time (indicated by dashed lines).

Figure 2A depicts the grand average of *error* and *hit* trials of all participants synchronized to throwing release. In *error* trials we found one sharp negative deflection in the EEG peaking 250 ms after ball release and another broader negative deflection reaching from about 350 ms after release until unambiguous visual KR. A small negative deflection was also visible in *hit* trials around 250 ms. Nevertheless, the mean amplitude of the difference wave in this time window differed significantly from zero (*t* = −2.5, *df* = 20, *p* = 0.02, Figure 2B). We also found a significant difference in the difference wave for the second deflection (*t* = −7.5, *df* = 20, *p* < 0.001, Figure 2B). After referencing the EEG epochs to the moment of KR feedback, the negative deflection in *hit* trials disappeared while both deflections in *error* trials were still present (Figure 2C). The difference wave deviated significantly from zero in the first time window (*t* = −2.6, *df* = 20, *p* = 0.02, Figure 2D) and in the second time window (*t* = −8.0, *df* = 20, *p* < 0.001, Figure 2D).

**Figure 3:**
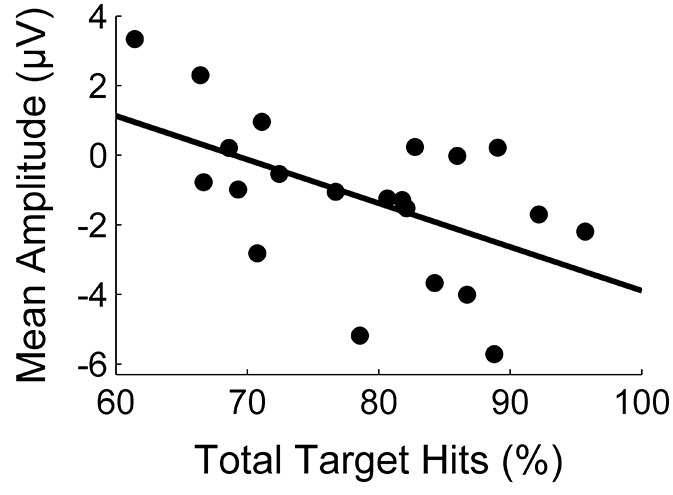
**Correlation between task performance (percentage of target hits) and the mean amplitude (μV) of the first deflection**.

The correlation between task performance and the amplitude of the first deflection is illustrated in Figure 3. The statistical analysis revealed a significant negative correlation (*r* = −0.54, *p* = 0.01). With respect to the second deflection, we similarly found a medium negative correlation which was, however, not significant (*r* = −0.35, *p* = 0.12).

## Discussion

The goal of the study was to quantify error prediction in a complex motor task via event-related potentials in the Electroencephalogram (EEG). We used a semi-virtual throwing task where the prediction about task success or failure was based on kinematics and dynamics of the movement. Participants had to hit a virtual target with a virtual ball by performing a throwing movement with a real lever. As result we found differences in the EEG for throws hitting the target relative to throws missing the target. In these error trials, we found a sharp negative deflection peaking 250 ms after ball release and another broader negative deflection reaching from about 300 ms after release until unambiguous visual KR (hitting or passing by the target). Since event-related potential analyses are based on averaging of several trials, the varying times between release and KR could have caused temporal smearing. To avoid false interpretations due to such smeared signals, we re-referenced the EEG segments to the moment of KR feedback. As a result, the two deflections were still present and can therefore be regarded as relatively robust error signals. In contrast, a small negative deflection in *hit* trials that was visible whit reference to release vanished after re-referencing to the moment of feedback.

Now, to interpret the two error signals it is important to understand that the present study combines two different error processing streams:

a. error processing based on prediction from active movement execution,
b. error processing based on passive action monitoring.

In *a*, the movement is executed and an error is predicted prior to any information about the movement outcome. We assume that this prediction is accomplished via a forward model of the task. Forward models-neural networks that model the input-output relations of the musculoskeletal plant-have long been postulated to be used in generating predictions about future states of the sensorimotor system given its current state and an outgoing motor command (Heuer, 1983; Miall and Wolpert, 1996; Kawato, 1999). An efference copy of the motor command is used to simulate, or in other words predict, the consequent sensory effects of the movement. In the throwing task, release angle and release velocity are the determining variables with respect to task success. According to shape and timing of the first error signal, we suppose that this first signal represents an error prediction process. However, we think that this prediction process is not merely based on efferent commands. The first error signal has an onset of on average about 200 ms after release. This time window may be sufficient to also use incoming visual and proprioceptive information about the movement execution (Jeannerod, 1988). There is evidence that humans integrate sensory feedback with the prediction about sensory consequences of actions to achieve a more accurate estimate of the actual state of the body and the environment (Wolpert et al., 1995; Desmurget and Grafton, 2000; Shadmehr et al., 2010). Thus, we interpret the first error signal as a result of scenario *a* where the error is predicted on the basis of efferent commands and afferent (proprioceptive and visual) information about the throwing movement (participants could see their arm as well as the virtual lever moving on the computer monitor).

In contrast, the second error signal might arise from processing stream *b*, concretely the monitoring of visual information about the ball flight. This processing stream can also apply when passively observing the ball flight without having actively executed the throw. Concretely, participants saw the complete ball flight after release on the computer screen and therewith the ball approaching the target. Thus, with increasing flight time, it becomes easier to extrapolate the ball trajectory and thus to estimate task success. We assume that the closer the ball comes to the target, the more this processing stream might resemble feedback processing. With the difference that the information about the result is not a binary signal conveyed at a single point in time after movement termination. It is rather an accumulation of certainties about whether the ball hits or misses the target. This accumulation might have given rise to the second error signal. In order to confirm these interpretations and to disambiguate the two error processing streams, error prediction and action monitoring need to be disentangled in further experiments.

Irrespective of the concrete functional significances of the two error signals, the study showed that also in a complex throwing task Ne/ERN-like components emerge prior to unambiguous feedback about the movement outcome (knowledge of result, KR). Hence, prediction contributes in any case at least partly to the generation of these components. Moreover, the signals occurred after movement termination. Thus, movement execution and potential corrective submovements can be excluded as contributors to the Ne/ERN. The study hereby extents the findings about the Ne/ERN in tasks with rather cognitive prediction demands and in continuous motor tasks with correction possibilities to tasks where the prediction is more of a motor nature and separated from movement execution.

Lutz et al. (2013), Peterburs et al. (2012), and Holroyd and colleagues (Holroyd and Coles, 2002; Nieuwenhuis et al., 2002; Holroyd et al., 2004) also conclude from their results that the prediction process contributing to the Ne/ERN is accomplished by a forward model. If this conclusion is true, it could reversely be used to gain information about the quality of the forward model. The theory of forward models postulates that an improvement of the forward model should result in faster learning (Jordan and Rumelhart, 1992). Participants in the present study differed in performance. Thus, assuming that these performance differences arose from different learning rates, they might be a function of the quality of the forward model. In consequence, one would expect a larger amplitude in the first error signal (assumingly representing prediction processes) in participants with high performance and* vice versa*. Mean amplitude of this signal and the percentage of target hits showed this correlation. We, therefore, suggest that task performance is positively correlated with error prediction and that this could be an indicator of the first error signal representing error prediction by means of forward processing. Perspectively, there are further studies necessary to confirm these interpretations. The prediction process needs to be completely separated from processes like action monitoring so as be able to clearly associate it to the underlying neural signals.

## Author Contributions

LM made substantial contributions to the conception and design of the work, the acquisition and interpretation of the work, drafted the work, approved the final version to be published and agreed to be accountable for all aspects of the work.

HMa made substantial contributions to the conception of the work, the interpretation of the work, revised the work, approved the final version to be published and agreed to be accountable for all aspects of the work.

HM made substantial contributions to the interpretation of the work, revised the work, approved the final version to be published and agreed to be accountable for all aspects of the work.

## Conflict of Interest Statement

The authors declare that the research was conducted in the absence of any commercial or financial relationships that could be construed as a potential conflict of interest.
